# Health system factors influencing uptake of Human Papilloma Virus (HPV) vaccine among adolescent girls 9-15 years in Mbale District, Uganda

**DOI:** 10.1186/s12889-020-8302-z

**Published:** 2020-02-04

**Authors:** Juliet Nabirye, Livex Andrew Okwi, Rebecca Nuwematsiko, George Kiwanuka, Fiston Muneza, Carol Kamya, Juliet N. Babirye

**Affiliations:** 1 Department of Health Policy, Planning and Management Makerere University School of Public Health College of Health Sciences, P.O. Box 7072, Kampala, Uganda; 2 Department of Disease control and Environmental Health, University School of Public Health College of Health Sciences, Kampala, Uganda; 3 Department of Biomedical sciences, Makerere University, School of medicine College of Health Sciences, Kampala, Uganda; 4 Department of Epidemiology and Biostatistics, School of Public Health, Makerere University, Kampala, Uganda

**Keywords:** Uptake, Health system, Human papillomavirus vaccine, Cervical cancer, Low income country, Adolescent girls

## Abstract

**Background:**

Globally, cervical cancer is the fourth most common cancer in women with more than 85% of the burden in developing countries. In Uganda, cervical cancer has shown an increase of 1.8% per annum over the last 20 years. The availability of the Human Papillomavirus (HPV) vaccine presents an opportunity to prevent cervical cancer. Understanding how the health system influences uptake of the vaccine is critical to improve it. This study aimed to assess how the health systems is influencing uptake of HPV vaccine so as to inform policy for vaccine implementation and uptake in Mbale district, Eastern Uganda.

**Methods:**

We conducted a cross sectional study of 407 respondents, selected from 56 villages. Six key informant interviews were conducted with District Health Officials involved in implementation of the HPV vaccine. Quantitative data was analyzed using Stata V.13. Prevalence ratios with their confidence intervals were reported. Qualitative data was audio recorded, transcribed verbatim and analyzed using MAXQDA V.12, using the six steps of thematic analysis developed by Braun and Clarke.

**Results:**

Fifty six (14%) of 407 adolescents self-reported vaccine uptake. 182 (52.3%) of 348 reported lack of awareness about the HPV vaccine as the major reason for not having received it. Receiving vaccines from outreach clinics (*p* = 0.02), having many options from which to receive the vaccine (p = 0.02), getting an explanation on possible side-effects (*p* = 0.024), and receiving the vaccine alongside other services (p = 0.024) were positively associated with uptake.

Key informants reported inconsistency in vaccine supply, inadequate training on HPV vaccine, and the lack of a clear target for HPV vaccine coverage as the factors that contribute to low uptake.

**Conclusion:**

We recommend training of health workers to provide adequate information on HPV vaccine, raising awareness of the vaccine in markets, schools, and radio talk shows, and communicating the target to health workers.

Uptake of the HPV vaccine was lower than the Ministry of Health target of 80%. We recommend training of health workers to clearly provide adequate information on HPV vaccine, increasing awareness about the vaccine to the adolescents and increasing access for girls in and out of school.

## Background

Globally, cervical cancer is the fourth most common cancer in women with more than 85% of the burden in developing countries [1]. The majority of cervical cancer mortality occurs in developing countries, where screening and optimal treatment are not adequately available [2]. Cancer of the cervix constituted 22.2% of all cancers among women in Sub-Saharan Africa in 2012 [3]. In Uganda, cervical cancer is the number one cancer killer disease among women, this is followed by breast cancer [4]. With the incidence standing at 52 /100,000 women of reproductive age, it is one of the highest globally. Regrettably, more than half of these women die every year [5, 6]. The Kampala cancer registry shows that Uganda has an age standardized incidence rate of 47.5 per 100,000 against the global estimate of 15.8 per 100,000 [7]**.** Many of the Cervical cancer cases present with an advanced stage of the disease [8].

Providing the Human Papilloma Virus (HPV) vaccine is aimed at primary prevention against cervical cancer so that there is no risk of infection progressing to cervical cancer later in life, because HPV is responsible for almost 90% of cervical cancer cases [9]. It is estimated that the HPV vaccine will reduce deaths from cervical cancer by two-thirds if uptake reaches 80% [10]. Two vaccines to prevent HPV infection, the cause of cervical cancer, are now approved for use in over 120 countries. This has created an opportunity to greatly enhance prevention of cervical cancer. The HPV immunization program is expected to have a significant impact on public health, however, challenges exist with delivery of the vaccine to adolescents aged 9 to 15 years which is the recommended population for HPV vaccinations by the World Health Organization [11]. This is because routine immunizations in most national programs target children younger than 5 years of age [12, 13]. The Ministry of Health Uganda in partnership with a drug manufacturing company in the United States of America (Merk Sharpe and Dohme) launched a vaccine program in 2012, targeting 140,000 pre-adolescents. The vaccine is relatively new in Uganda, it is given out free of charge but uptake has remained low in many districts with national average estimated at 17% as of December 2016 [14]. Delivering HPV vaccines to young adolescents therefore requires a different kind of health programming [15]. Since this is a new vaccine, very few studies have been conducted about factors influencing uptake of the vaccines, therefore this study aimed to assess how the health system is influencing uptake of the HPV vaccine for adolescents 9–15 years so as to inform HPV vaccination policy and implementation program in Uganda.

## Methods

### Study design and population

We conducted a cross-sectional study in Mbale district in Eastern Uganda. Mbale district has a population of 488,990 people of which 52.3% are female, and 21% are between 10 and 17 years of age. The district was among the first districts where the HPV vaccination program was first implemented in 2012. We used a structured questionnaire to interview the adolescent girls. we held six key informant interviews with health workers in the district. The quantitative and qualitative data collection methods helped to obtain convergence and substantiation among the different health system factors. The multiple perspectives aimed to provide an opportunity to develop a more complete understanding of the health system factors influencing HPV vaccine uptake.

### Study population

The study enrolled female adolescents aged 9–15 years because they were expected to be in Primary four or within the expected age group for the vaccination schedule.

### Sampling

Quantitative data were selected using a structure questionnaire, in a multi stage cross sectional design. We used Bennett’s cluster survey sampling formula taking an assumption of a prevalence of 50%, a precision of 0.032 [16] and a margin of error of 5%. The sample size was 392 respondents. On adjusting for non-response, at a rate of 10%, the final sample size was 431 respondents. The study used a three-stage sampling procedure; in the first stage, we randomly selected five sub-counties out of the twenty in the district. In this study, a cluster was equivalent to a village. We randomly selected five sub-counties out of the twenty-three and from each sub county, we selected two parishes to give a total of ten parishes. A list of all villages from the selected parishes was then used to randomly select the total of 56 villages. We then interviewed seven adolescents 9–15 years, eligible for the HPV vaccine from each village using the Village Health Team’s (VHT) guide, and taking only those who were residents of the selected villages in Mbale district for at least 2 years. A consideration of 2 years was taken because the national rollout of the vaccine was done in 2015. Care takers and adolescents who were not found in their homes after three consecutive visits were replaced with the next household. If a care taker was too ill to take the interview, they were excluded and replaced.

Health system factors were assessed through key informant interviews and an observation checklist. We conducted six key informant interviews with the district health team members who had an expert opinion about the health services factors that influence uptake of HPV vaccination in the district. The district team members included the following: the District Health Officer for maternal and child health, the District Cold Chain Technician and health facility In-charges. The numbers of Key Informant Interviews were deemed sufficient when additional interviews yielded little new information on the core study objectives. The interviews were audio recorded after informed verbal consent was obtained from the participants. We observed for key vaccines, supplies in selected health facilities within the sub-counties using the World Health Organization (WHO) checklist for vaccines and supplies.

### Dependent and independent variables

The dependent variable was uptake of the HPV vaccine, this was measured by having a vaccination card that indicates the number of doses attained and recall of obtaining an injection on the left upper arm if the child was between 9 and 15 years. Initiation was defined as having received at least one of the recommended two dose series of the HPV vaccine and Uptake was defined as completing the two doses of the HPV vaccine.

### Data analysis

Quantitative data were entered into Excel 2010, and then exported to Stata Version 13 for statistical analysis. The data were97 summarized into frequencies and proportions for categorical variables and mean. At bivariate level of analysis, Prevalence Ratio (PR) measure was used to assess relationship between the dependent variable (HPV vaccine uptake) and the independent factors. The prevalence ratios were computed using a generalized linear model with Poisson family and a log link with robust errors. At multivariable analysis, all the independent factors with a *P* value less than 0.15 at bivariate analysis were included in the multivariable model to obtain the adjusted Prevalence ratios. The backward elimination approach was used to obtain the best model with the log likelihood that was closer to zero. The significance level for all the analysis was set at *P* ≤ 0.05. The model comprised of age group, tribe, religion, and occupation, having many options from which to receive the HPV vaccine, knowing where to report side effects, having received any other vaccines, getting HPV vaccine together with other services, knowing where to report the side effects, and receiving adequate information about the vaccine.

For qualitative data, audio tape recordings were all together transcribed verbatim, coded and uploaded qualitative data analysis software MAXQDA version 12. Recurring themes were identified within and between each interview [17]. Two independent researchers were involved in coding. These transcripts were scrutinized to ensure reliability in the use of codes between the coders. The independent lists of codes were reviewed to assess inter-coder agreement. Discrepancies were clarified and resolved by comparing each coder’s results with raw data until consensus was reached. A list of codes was then finalized. The codes were based on the study objectives.

Data was then condensed through expressive, text-based summaries and data display matrices. The matrices facilitated to distinguish among the themes and groups. Quotes were then selected that were representative of the main themes.

## Results

A total of 407 respondents were interviewed from the calculated sample size of 432 giving a non-response rate of 9%.

### Socio-demographic characteristics of the respondents

Table 1 shows the baseline characteristics of the respondents; the mean age was 11.8 ± 1.8 years with a minimum age of 9 years and a maximum of 15 years. 75% (306/407) of the respondents lived in rural areas. The care takers of the adolescents were mostly married (73.5%) with half of them having attained up to primary level of education (50.1%). Most of the respondents were of the Gishu tribe (71.3%), and more than two thirds were of Muslim faith (41.5%). Most (71%) of the respondents lived approximately 1 km to 3 km from a health facility.

**Table 1:** Shows the socio demographic characteristics of the participants

Characteristic	Frequency (*N* = 407)	Percentage (%)
Age (years)		
9–10	116	28.5
11–12	130	31.9
13+	161	39.6
Residence		
Urban	101	24.8
Rural	306	75.2
Marital status of caretaker		
Single	18	4.5
Married	299	73.5
Divorced/separated	49	12.0
Widowed	41	10.0
Education level of caretaker		
No formal education	46	11.3
Primary level	204	50.1
Secondary level	138	33.9
Tertiary level	19	4.7
Religion		
Catholic	75	18.4
Anglican	129	31.7
Moslem	169	41.5
Others	33	8.4
Tribe		
Bagishu	290	71.3
Banyole	62	15.2
Others	55	13.5
Occupation of care taker		
House wife	59	14.5
Formal employment	22	5.4
Business women	131	32.2
Farmer	195	47.9
Number of siblings		
One to two	61	15.0
Three to four	192	47.2
Five and more	154	37.8
Distance to health facility		
Less than 1 km	78	19.2
Between 1 and 3 km	289	71.0
Greater than 3 km	40	9.8

### Initiation of the HPV vaccine

**Fig. 1:**
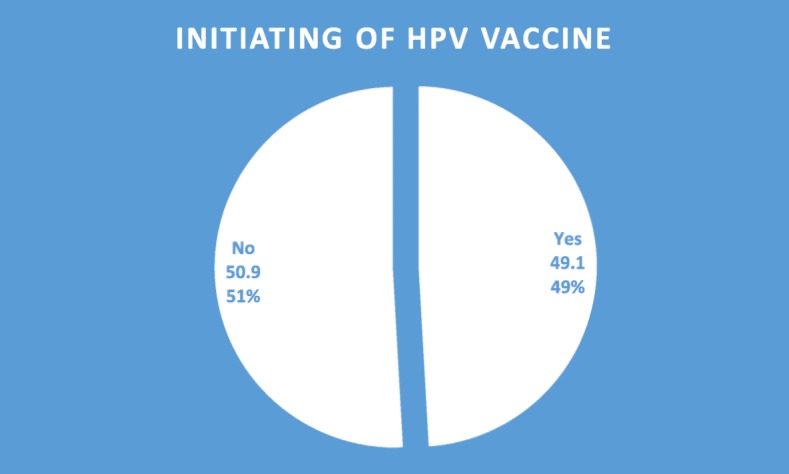
Bar chart showing initiation of the HPV vaccine among adolescent girls

Figure 1 shows the total number of adolescents interviewed, 49% (200/407) had initiated the vaccination, of these, adolescents that had initiated the HPV vaccine, 13.8% (56/407) had received both doses and thus completed the vaccination. See Fig. 1.

Table 2 shows the main reasons for not receiving the vaccine. The total number of respondents was 348 because it included only those who had either received one dose or none. Lack of awareness was the main reason given by 45% (182/348) of the adolescents. While some respondents mentioned that they were not aware about the HPV vaccine, some were not aware of the number of doses that they must receive and others were not aware of the schedule or interval of the vaccines. Less than 2% (6/348) of the respondents mentioned unfriendly health workers as a major reason for failure to obtain the vaccine, while 4 % 4% (14/348) of the respondents who had received one dose were aware that they were due for a second dose. Other reasons for not vaccinating include reluctance to vaccinate, being afraid of vaccines, and myths about the vaccines.

**Table 2:** Major reasons for failing to obtain (initiate and or complete) the HPV vaccine

Reason	Frequency (*N* = 348)	Percentage
Distance to health facility	50	14.4
No money	35	10.0
Unfriendly health workers	6	1.7
Lack of awareness	182	52.3
Not due for second dose	14	4.0
Others	61	17.5
Total	348*	100

*Total is more than the number of those who have not got the HPV vaccine since even those who had got one dose were asked the question

### Factors associated with uptake of the HPV vaccine

Table 3 shows that the prevalence of uptake was two times higher among the age group of 11 to 12 years (PR 2.1, 95% CI 1.0–4.4) compared to those who are 9 to 10 years. It was also twice higher among the Banyole ethnic group (UPR2.2, 95% CI, 1.18–4.04) compared to the Bagishu, it was also six times higher among adolescents whose care takers were business women (unadjusted PRR 5.9, 95% CI 2.0–16.9) compared to those who were housewives.

**Table 3:** Bivariate analysis of independent factors associated with HPV vaccine uptake among adolescents

Variables	Received two doses of		UPR (95% Confidence Interval)	*P*-value
	HPV vaccine			
Education level of caretaker	Yes (%)	No (%)		
None	6(10.7)	40(11.4)	1.0	
Primary	18(32.1)	186(53)	0.7(0.27–1.70)	0.407
Secondary & above	32(57.1)	125(35.6)	1.6(0.65–3.73)	0.316
Age group				
9–10	9(16.1)	107(30.5)	1.0	
11–12	33(58.9)	165(47.0)	2.1(1.0–4.4)	0.042*
13–15	14(250.)	79(22.5)	1.9(0.84–4.4)	0.121
Religion				
Catholics	13(23.2)	62(17.7)	1.0	
Anglicans	24(42.86)	105(29.9)	1.1(0.55–2.10)	0.837
Muslims	14(25.0)	155(44.2)	0.5(0.22–1.01)	0.055
Others	5(8.9)	29(8.3)	0.9(0.30–2.38)	0.755
Currently in school				
Yes	52(92.9)	323(92.0)	1.1(0.4–3.04)	0.842
No	4(7.1)	28(7.98)	1.0	
Nature of the school				
Government aided	15(28.3)	240(73.4)	1.0	
Private funded	38(71.7)	87(26.6)	1.1(0.59–.955)	0.811
Tribe				
Bagishu	32(57.1)	258(73.5)	1.0	
Banyole	15(27)	47(13.4)	2.2(1.18–4.04)	0.012*
Others	9(16.1)	46(13.1)	1.5(0.707–3.10)	0.296
Occupation				
Housewife	5(8.9)	54(15.4)	1.0	
Business woman	11(19.6)	11(3.1)	5.9(2.049–16.9)	< 0.001**
Formal employment	15(26.8)	116(33.1)	1.4(0.49–3.72)	0.56
Farmer	25(44.6)	170(48.4)	1.5(0.579–3.95)	0.398
Distance to the health facility				
Less than 1 km	17(30.4)	61(17.4)	1.0	
1 km -3 km	34(60.7)	255(72.7)	1.9(0.30–0.97)	0.038*
More than 3 km	5(8.9)	35(9.8)	1.8(0.21–1.6)	0.274
Obtained HPV vaccine from an outreach clinic				
Yes	47(83.9)	121(34.5)	7.4(3.6–15.2)	< 0.001**
No	9(16.1)	230(65.5)	1.0	
Received an explanation on side effects of vaccine				
Yes	45(80.4)	68(19.4)	10.6(5.5–20.6)	< 0.001**
No	11(19.6)	283(80.6)	1.0	
Paid for HPV vaccine?				
Yes	4(7.1)	11(3.1)	2.0(0.72–5.5)	0.178
No	52(92.9)	340(96.9)	1.0	
Had many options from where to receive HPV vaccine				
Yes	44(81.5)	107(31.3)	7.1(3.5–14.2)	< 0.001**
No	10(18.5)	235(68.7)	1.0	
Received any other childhood vaccines				
Yes	28(50)	118(33.6)	1.8(1.05–3.0)	0.030*
No	28(50)	233(66.4)	1.0	
Received HPV vaccine alongside other services				
Yes	26(46.4)	27(7.7)	5.8(3.4–9.7)	< 0.001**
No	30(53.6)	323(92.3)	1.0	
Received adequate information about the vaccine				
Yes	35(62.5)	53(15.1)	6.0(3.5–10.4)	< 0.001**
No	21(37.5)	298(84.9)	1.0	
Heard of someone with side effects of HPV vaccine				
Yes	5(8.9)	9(2.6)	2.8(1.0–6.8)	0.031*
No	51(91.1)	342(97.4)	1.0	
A of where to report side effects of HPV vaccine?				
Yes	20(35.7)	44(12.5)	3.0 (1.7–5.1)	< 0.001**
No	36(64.3)	307(87.5)	1.0	

*CI* Confidence interval, Significance level *P* < 0.05, *P < 0.01, **P < 0.001

Uptake for the vaccine was also twice higher among those who had received other childhood vaccines (UPR 1.8, 95% CI 1.05–3.01), and seven times higher among those who obtained HPV vaccine from outreach clinics (UPR 7.4, 95% CI 3.6–15.15). Additionally, uptake was eleven times higher among those who received an explanation on the side effects of the HPV vaccine (UPR 10.6, 95% CI 5.5–20.57), six times higher among those who got the vaccines alongside other services (unadjusted PRR 5.8, 95%CI 3.4–9.7), seven times higher among adolescents who had many options from where to receive HPV vaccine (unadjusted PRR 7.1, 95% CI 3.5–14.18) and three times higher among those with knowledge of where to report side effects (UPR 3.0 95% CI 1.7–5.1).

Table 4; shows the multivariable analysis, after adjusting for possible confounders, the prevalence of uptake of the HPV vaccine was two and a half times higher among girls who had received the vaccine from an outreach clinic APR 2.6,95% CI: 1.2–5.9) compared to those who obtained from static sites. It was also three times higher among those who received an explanation for possible side effects (APR 2.7, 95% CI 1.1–6.4) compared to those who didn’t get an explanation. Prevalence was also twice higher among adolescents who received vaccines together with other services (APR 2.3, 95% CI 1.1–4.6) and four times more among adolescents who had many options from where to receive the HPV vaccine (APR 3.6, 95% CI 1.6–8.1) after controlling for all the other significant variables at bivariate analysis. See table below.

**Table 4:** Multivariable analysis of independent factors associated with HPV vaccine uptake among adolescents

Variables	*N* = 407 (%)	UPR (95% CI)	Adj.PR(95%CI)	*P*-value
Age group				
9–10	116(28.5)	1.0	1.0	
11–12	198(48.6)	2.1(1.0–4.4)	1.3(0.57–3.01)	0.527
13–15	93(22.9)	1.9(0.84–4.4)	1.2(0.48–3.27)	0.64
Obtaining vaccine from outreach clinic				
Yes	168(41.3)	7.4(3.6–15.15)	2.6(1.16–5.86)	0.020*
No	239(58.7)	1.0	1.0	
Explanation on side effects of HPV vaccine				
Yes	113(27.8)	10.6(5.5–20.57)	2.7(1.13–6.4)	0.024*
No	294(72.2)	1.0	1.0	
Many options to receive vaccine				
Yes	151(38.1)	7.1(3.5–14.18)	3.6(1.58–8.13)	0.002*
No	245(61.9)	1.0	1.0	
Received other vaccines				
Yes	146(35.9)	1.8(1.05–3.01)	0.6(0.317–1.2)	0.206
No	261(64.1)	1.0	1.0	
Got vaccine with other services				
Yes	53(13.0)	5.8(3.4–9.7)	2.3(1.11–4.59)	0.024*
No	353(86.7)	1.0	1.0	
Received adequate information about HPV vaccine				
Yes	88	6.0(3.5–10.4)	1.6(0.85–3.33)	0.137
No	319	1.0	1.0	
Heard of someone with side effects of vaccine				
Yes	14	2.8(1.0–6.8)	1.2(0.38–3.9)	0.723
No	393	1.0	1.0	

### Barriers to service delivery

In the study, the major barriers to service delivery from the key informant interviews included low financing, myths about the vaccine, unclear communication on the target for the vaccine’s coverage and transport challenges to reach the adolescents in the community. Funding for immunization activities was previously provided by other organizations that supplemented the Primary Health Care (PHC) funds but this was not happening at the time of the study. This is affirmed by one key informant who states that;

*“Previously, GAVI was supplementing the PHC Funds but in the last financial year, it has been hard to manage and I am sure that some facilities have not been able to conduct outreaches in both the schools and the community” (Key informant 2, DHT).*

Some key informants revealed that private schools and private health facilities are not given the HPV vaccine and this creates inequity in access for those who prefer to utilize private health facilities for receipt of the vaccine and girls in private schools.

*“We supply the vaccine to the public and private not for profit health facilities, we are not giving the private clinics, this is because many of them are not equipped with the cold chain and they do not report to us.”*

*(Key Informant 1, DHT)*

*“We give out this vaccine to government schools only, the private schools don’t benefit because they have to obtain parental consent for their pupils to get it. In the Government schools, the school authority gives the consent”.*

*(Key Informant 3 health facility in-charge)*

### Facilitators to service delivery

HPV vaccine delivery has been made easier through the school-based delivery approach because the target group was clear but there was confusion as to whether to vaccinate those in primary five since the target class is primary four. With the school-based approach, health facilities have been able to liaise with schools to make it easy for the adolescents to receive HPV vaccine as stated by one key informant below.

*“Health facilities liaise with the schools so that arrangements are made for the HPV vaccination, for example they set aside a classroom where the equipment can be placed so that the vaccination can take place”.*

(*Key Informant 2, Healthy facility in-charge*)

### Barriers for human resources for health

The major barriers to human resources for health mentioned were the inadequate staff to run the work in the health center and insufficient training on HPV vaccine.

*“We have few staff, which also compromises our service delivery. If some health workers go to the outreach clinic, you can feel the impact in the health facility when a few of us are left here”.*

(*Key Informant 1, Healthy facility in-charge*)

Despite the inadequate staff at the health facility, the VHTs and other community mobilisers support the health workers in mobilizing the community to take their daughters for vaccination thus motivating them. In addition, health workers were motivated to work with the available Primary Health Care funds. The team work and role played by the Village health teams and other mobilisers in the community motivated them.

“*PHC funds have helped to facilitate vaccinators and this is a good strategy for us. In addition, we use phone messages to thank them for the good work they do despite the hardship”* (*Key informant 1, District Health Team*).

### Barriers to vaccines, supplies and medicines

Inconsistency in vaccine supply was noted in both the checklists and from various key informants and records in health facilities; the first supply of vaccines doses was underestimated.

*“The inconsistence in vaccine supply is a major barrier to completion of the doses, and it is something that I know is beyond the District Health Office to handle.”*

*(Key informant 1, DHT)*

*“Supply of the vaccine is very poor and inconsistent. Despite this, we give out the doses as and when we receive the stock, but in that case, we can’t ascertain the efficacy of the vaccine”.*

*(Key informant 2, DHT*)

The integration of the HPV vaccines with other services such as child days plus helps to increase coverage by taking advantage of the existing infrastructure to provide the vaccine. This is expressed by some key informants.

*“Furthermore, this is an integrated service and people get very many services at once, may be this has contributed to the success”.*

*(Key informant 4, Health facility in-charge)*

## Discussion

The study estimated the level of uptake for the bivalent HPV vaccine in Mbale district, in eastern Uganda and found that 14% of the study participants were fully vaccinated. However, the estimated uptake in this survey was lower than what the district reports for HPV uptake of 32%. This discrepancy may be due to errors in reporting from various health facilities into the Health Management Information System [18, 19]. The variance may also be due to unreliable census figures, an unclear denominator due to the stringent eligibility criteria for adolescent girls who should receive the HPV vaccine, the uptake was similar to a study done in Lira district, Northern Uganda which found that 14% of the adolescents were fully vaccinated [20].

The low uptake of the HPV vaccine was attributed to inadequate training among health workers about the vaccines; Uganda merged the measles campaign, Polio Supplementary Immunization Activities (SIA) and HPV vaccine introductory activities due to limited bandwidth within the Uganda National Expanded Program on Immunization (UNEPI) and insufficient funds to cover all activities. However, this led to key critical shortfalls in HPV implementation: training of health workers on HPV vaccine was reduced from 3 days to 1 day; and there was no social mobilization messaging on HPV vaccine because the vaccine had not yet arrived in the country, hence demand couldn’t be increased yet the vaccine was not immediately available.

Additionally, low uptake may also be attributed to the lack of Information, Education and Communication (IEC) materials on HPV vaccine in health facilities, schools and other communal places such as markets. These IEC materials are usually a way of communicating health related information to a vast majority of the population, this is in agreement with findings from one study which stressed the lack of education material on HPV vaccination given by health professionals to young adolescents as a barrier to vaccine uptake and emphasized the need to improve education about cervical cancer, prevention and HPV vaccination [21]. Several studies have highlighted the need for health workers to be trained to provide adequate information about this vaccine. In many of these studies, health workers are the most preferred source of information and influence the decision to vaccinate [22–24]. Furthermore, receiving adequate information from a health care provider greatly improved uptake of the vaccine, this finding is similar to other studies, where healthcare professionals impacted the choice for adolescents to receive the HPV vaccine [25, 26] and these decisions were shaped by confidence in the vaccination program and healthcare providers. This may call for health workers to provide a brief discussion on the vaccine, its benefits and possible side effects prior to administration [25].

### Brief explanation about the vaccine

An interventional study conducted in the United States of America showed the effect of a brief (10 minutes) group HPV educational session on knowledge and intent to vaccinate among young adults. Individuals in the intervention group were three times more likely to take on the vaccine [27, 28]; this is similar to findings from this study where girls who received an explanation on the side effects of the vaccine were almost three times more likely to take on the vaccine as compared to those who didn’t receive an explanation. Findings from this study show that adolescents who received adequate information about HPV vaccine were more likely to receive it, and this is similar to findings from another study conducted in Kenya which discovered that perceiving oneself to be adequately informed was a strong determinant of HPV vaccine uptake [29]. This means that health workers need to be trained to provide the necessary knowledge on HPV vaccine prior to provision on the vaccine to the adolescent girls.

### Low awareness among the target beneficiaries and caretakers

Lack of awareness was another major factor influencing initiation and uptake of the HPV vaccine, this finding is similar to a study which sought to understand suboptimal HPV vaccine uptake among ethnic minority adolescents, with the strongest predictor of initiation reported as vaccine awareness [30, 31]. The study also highlighted that the lack of information about HPV vaccine and where to obtain it by mothers negatively influenced their decision making [30]. Additionally, a study among women in Malawi, showed that respondents believed that HPV vaccine uptake would be increased if information were dispersed throughout the community, since they strongly believed that this would address the challenge of low awareness on HPV vaccine [32]. This shows the importance of social mobilization especially for new vaccines that are outside of the known target age group.

### Inadequate human resources for health

This study also found that human resources for health were inadequate in various health facilities to provide HPV vaccine. This may be due to the inadequate staffing levels at the district which is estimated at 73% at the time of the study. Health workers usually have to leave the health facility and move to schools to provide the HPV vaccine to the girls; this leaves the work at the health facility to a few health workers and increases the burden on the few staff members who remain at the facility. This finding is similar to one study that looked at uptake of HPV vaccine in low and middle income countries and also revealed that human resources for health were inadequate for HPV vaccine delivery [33–35]. The implication of this is that the Ministry of Health will need to find more innovative ways of increasing the human resource needed to provide the vaccine to this special age group. The insufficient human resources was reported as a challenge to vaccine delivery, this is consistent with findings from another study where human resources find it a challenge to go to outreach clinics, they use “vaccinators” to help ease on the work load [33].

### Vaccine and supplies

The availability of HPV vaccines was mentioned throughout interviews as having influence over adolescent girl’s uptake of HPV vaccine. As noted among the key informants, many times, the adolescents found the vaccine out of stock, and this worried the health workers about the efficacy of the vaccine since the second dose was received much later than the recommended time of 6 months interval. This finding is similar to a study in Malawi that elaborated the vaccine stock outs and inconsistency in supply as barriers to uptake [32].

The inconsistency in supply may be due to the fact that new vaccines impose pressure on the health systems of most developing countries. As a result, they are faced with challenges in their vaccine supply and logistics systems [36]. Additionally, storage capacity bottlenecks can occur at national, regional, and district levels and system inefficiencies threaten vaccine access, availability, and quality. At the national level, HPV forecasts and supplies were adjusted to cater for peak demand during the months of April and October while at district level, due to the limited knowledge of target age group, health facilities forecasts and deliveries did not align with monthly need including peak periods resulting in surplus in supply in some health facilities and stock-outs in others.

As Uganda adopts the HPV vaccine, the health system must attempt to reach people at different ages and in new settings, as a result, the logistics systems must be strengthened and improved.

### Service delivery

Integration of services was found to significantly increase uptake of the HPV vaccine. This is because adolescents get extra services such as deworming, family planning, HIV testing and health education that are given at these outreach clinics. Gavi recommends that integrated programs offer opportunities for other age-relevant services such as de-worming and nutritional supplements. This integrated approach presents an opportunity to reduce the cost and burden on health systems of delivering separate interventions [37].

This study found the cold chain (fridges, thermometer, vaccine carriers, and storage space) and the infrastructure was adequate, this is contrary to findings from two previous studies, where infrastructure for the delivery of the HPV vaccine was found to be lacking [33, 38]. The cold chain may have been found to be better due to the continuous support of the United Nations children’s fund (UNICEF) and Gavi support to the Expanded Program on Immunization in the district. There has also been an improvement in infrastructure because of continuous and more preparation for cold chain due to the introduction of Pneumococcal Conjugate Vaccine (PCV), and the change from Oral Polio Vaccine (OPV) to Injectable Polio Vaccine (IPV) plus other planned new vaccine introductions.

The similarly striking low rates of HPV vaccination in a study among Cambodian American teenagers highlighted the need to improve vaccination outreach [39];. These findings are similar to this study and thus can be used to develop targeted public health HPV vaccination programs for various geographical groups, which will reduce cervical cancer disparities. Outreach clinics are suitable, particularly for children out of school.

This study revealed that cost was not a barrier to obtaining the vaccine, this is contrary to another study where cost was a barrier to vaccination [40]. The difference in the findings may be due to the fact that the HPV vaccine is provided free of charge by the Government of Uganda and subsidized by the Global Alliance for Vaccines and Immunization [41]. One more study conducted on predictors of HPV vaccination among daughters of low-income Latina mothers identified independent predictors of HPV vaccine uptake, and low worry about how to pay for the vaccine was a predictor for vaccination [42].

### Study limitations and strengths

This study was cross-sectional in nature and therefore we cannot infer a causal relationship between awareness of the vaccine and subsequent uptake. This means that this study cannot depict that awareness on the HPV Vaccine always results in uptake. Nonetheless, the study addressed one of the key areas which is salient in the effort needed to enhance HPV Vaccine uptake in the country.

## Conclusion

Uptake for the HPV vaccine in this study was defined as completing two doses of the vaccine. In this study, uptake was 14%. This is much lower than the 80% national HPV vaccine coverage target. Lack of awareness about HPV vaccine was found to be the main reason for the low uptake of the HPV vaccine. Besides, lack of communication and advocacy on the vaccine to raise awareness also hampered its uptake.

Factors that positively influenced uptake of the vaccine include receiving an explanation for possible HPV vaccine side effects, having many options from where to get the vaccine, getting the vaccine from an outreach clinic, and getting the vaccine alongside other services.

### Recommendations

The Ministry of Health and implementing partners must aim at raising awareness about the HPV vaccine as a primary preventive mechanism against cervical cancer through various forms of media. In addition, the Government must nurture a public private partnership to include private health facilities in providing the HPV vaccine so as to increase coverage in areas that are mainly served by the private health services.

The District Health Team should conduct continuous on job training of health workers on HPV vaccine so that they can deliver quality information to the adolescents prior to receipt of the vaccine.

The Ministry of Health must ensure that the expected target for coverage is communicated to all relevant stakeholders so that they work towards it and are capable of monitoring and evaluating their work.

### Implications

Awareness can be improved by engaging various media options for the particular age group, communicating with the care takers of the children to allay fears that may lead to hesitancy and wide distribution of the vaccine to reach as many adolescents as possible.

## Data Availability

The dataset used and analyzed during this study is available from the corresponding author upon reasonable request.

## Abbreviations

APR: Adjusted Prevalence Ratio
DHO: District Health Officer
DHT: District Health Team
GAVI: Global Alliance for Vaccines and Immunization
HPV: Human Papilloma Virus
IEC: Information, Education and Communication
PHC: Primary Health Care
PR: Prevalence Ratio
SIA: Supplementary Immunization Activities
UNEPI: Uganda National Expanded Program on Immunization
UPR: Unadjusted Prevalence Ratio
VHT: Village Health Team
WHO: World Health Organization
