# Correction to: Health system factors influencing uptake of human papilloma virus (HPV) vaccine among adolescent girls 9–15 years in Mbale District, Uganda

**DOI:** 10.1186/s12889-020-8348-y

**Published:** 2020-04-15

**Authors:** Juliet Nabirye, Livex Andrew Okwi, Rebecca Nuwematsiko, George Kiwanuka, Fiston Muneza, Carol Kamya, Juliet N. Babirye

**Affiliations:** 1 Department of Health Policy, Planning and Management Makerere University School of Public Health College of Health Sciences, P.O. Box 7072, Kampala, Uganda; 2 Department of Disease control and Environmental Health, University School of Public Health College of Health Sciences, Kampala, Uganda; 3 Department of Biomedical sciences, Makerere University, School of medicine College of Health Sciences, Kampala, Uganda; 4 Department of Epidemiology and Biostatistics, School of Public Health, Makerere University, Kampala, Uganda

**Correction to: BMC Public Health (2020) 20:171**

**https://doi.org/10.1186/s12889-020-8302-z**

In the original article [1] the first paragraph of the Background section was omitted due to a discrepancy between the metadata of the article and the PDF version. This Correction article shows the missing paragraph of the Background section. The original article has been updated.

## **Background**

Globally, cervical cancer is the fourth most common cancer in women with more than 85% of the burden in developing countries [1]. The majority of cervical cancer mortality occurs in developing countries, where screening and optimal treatment are not adequately available [2]. Cancer of the cervix constituted 22.2% of all cancers among women in Sub-Saharan Africa in 2012 [3]. In Uganda, cervical cancer is the number one cancer killer disease among women, this is followed by breast cancer [4]. With the incidence standing at 52 /100,000 women of reproductive age, it is one of the highest globally. Regrettably, more than half of these women die every year [5, 6]. The Kampala cancer registry shows that Uganda has an age standardized incidence rate of 47.5 per 100,000 against the global estimate of 15.8 per 100,000 [7]. Many of the Cervical cancer cases present with an advanced stage of the disease [8].
